# Molecular Dynamics—From Small Molecules to Macromolecules

**DOI:** 10.3390/ijms22073761

**Published:** 2021-04-05

**Authors:** Ki Hyun Nam

**Affiliations:** Department of Life Science, Pohang University of Science and Technology, Pohang 37673, Korea; structures@postech.ac.kr

**Keywords:** molecular dynamics, time-resolved, structural change, conformational change

## Abstract

All molecular systems, from small molecules to macromolecules, exhibit specific characteristics for a specific environment and time. In order to gain an accurate understanding of the functions of all types of molecules, studies of their structure and dynamics are essential. Through dynamic studies, using techniques such as spectroscopy, structure determination, and computer analysis, it is possible to collect functional information on molecules at specific times and in specific environments. Such information not only reveals the properties and mechanisms of action of molecules but also provides insights that can be applied to various industries, such as the development of new materials and drugs. Herein, I discuss the importance of molecular dynamics studies, present the time scale of molecular motion, and review techniques for analyzing molecular dynamics.

Imagine this example of why molecular dynamics research is important. There is an advertisement for a new vehicle in the newspaper. It is built primarily for driving on the road like a car, but it can fly like a light aircraft and can be sailed like a ship on rivers or seas. Of course, it is equipped with fully autonomous driving capability, and powered by sustainable energy. Below the introduction to each of these versatile features is a picture of the vehicle engaged in each mode of transport (ground, air, and sea). We can extrapolate a lot from these three pictures by imagination, but some key questions remain unanswered. How will this vehicle transform from one mode of transport to another (i.e., car to plane to ship)? How long will each transformation take? How long can this transport run without replacing its energy source? How perfect/safe is its autonomous driving? How does it handle traffic conditions on the road, sky, and sea? We cannot say anything with confidence with just three pictures. Each mode of transport will have its own operating characteristics, and these characteristics will also change in adaptation to changes in operating environment. We will need tons of pictures and/or intelligently produced videos to accurately understand the vehicle’s characteristics under all conditions we may encounter in each mode of transportation. With continuous information (as is provided by videos), we can accurately understand the vehicle’s performance, whereas fragmented information (such as that from pictures) is insufficient to understand the new vehicle.

This transportation-based analogy illustrates the importance of continuous observation, like movies, for the understanding of complex processes such as those studied in the sciences. In nature, there are many molecules (ranging from small molecules to macromolecules) with interesting and important molecular properties that can be elucidated using various scientific techniques.

Among these, techniques for obtaining high-resolution structural information are essential as they provide intuitive insights into important structural and functional characteristics of molecules that play important roles in life. Currently, the three dimensional structures of small molecules can be obtained from repositories such as the Cambridge Structural Database (CSD) [1], which contains 1,123,962 small-molecule organic or metal-organic structures obtained using X-ray, neutron diffraction, or microcrystal electron diffraction (MicroED) techniques [2]. The three dimensional structure of macromolecules can be obtained from repositories such as the Protein Data Bank (PDB) [3] and Electron Microscopy Data Bank (EMDB), containing 175,759 and 14,493 macromolecular structures (protein, DNA, RNA, virus, or protein-nucleic acid complexes), respectively, determined using X-ray, nuclear magnetic resonance (NMR), cryogenic electron microscopy (Cryo-EM), or MicroED techniques. These structures are very useful for describing molecular functions and features and are applied as inputs for computational analysis models such as molecular dynamics (MD) simulation [4]. However, since most of this structural information mainly deals with static information in a specific state, it cannot provide accurate information on structural changes caused by external environments or stimuli. Interpretation of a molecule’s function using static information cannot explain the complete underlying mechanism and can be misleading [5]. In order to understand the exact function of a molecule, it is necessary to obtain dynamic information about the molecule in a spatiotemporal context [6], as the molecular functions are intimately related to structure and dynamics [7]. In order to observe the dynamics of a molecule, it is important to understand the time scale of the motion of the target molecule (Figure 1). The time scales of molecular motion such as electronic motion, photodissociation, photoionization, bond vibrations, molecular collisions, vibration relaxation, solvation, proton transfer, temperature jump Raman, fluorescence, molecular rotations, large molecule relaxation, molecular diffusion, and phosphorescence motion varies between a few seconds to femtoseconds [8,9,10]. When observing the dynamics of a molecule, the movement time scale of the molecule must be considered and an appropriate experimental technique must be applied to observe it.

Various techniques such as spectroscopy, structure determination and computer analysis are applied to the study of molecular dynamics (Figure 2). In order to observe the dynamics of a molecule at a specific time, it is necessary to observe the molecule at much shorter time intervals. In this context, ultrafast lasers have a shorter pulse duration than most characteristic relaxation times of the condensed phase, making it possible to carry out detailed characterization of the temporal, spatial, and spectral properties of materials [11]. The development of femtosecond spectroscopy ushered in the era of real-time investigation of the motion of atoms in solid, liquid, and gaseous state molecules [12,13]. Furthermore, advances in time-resolved spectroscopy allows us to visualize the dynamics of the various types of atoms and molecules at dynamic time units (up to tens of femtoseconds) [12]. Time-resolved vibrational spectroscopies, such as infrared absorption spectroscopy (time-resolved IR spectroscopy) and Raman scattering (time-resolved Raman spectroscopy), have been widely applied as a solution for studying molecular dynamics [14].

Meanwhile, techniques such as X-ray diffraction/scattering, NMR, Cryo-EM, MicroED, and neutron scattering/diffraction are widely applied to study the mechanism of action by revealing the structure of a molecule [2,15,20]. These techniques enable the study of the flexibility of a molecule within a single static structure, and they provide dynamic information by collecting data regarding external stimuli/environment or other static data in a complex. With the development of X-ray technology, an X-ray free electron laser (XFEL) with ultrashort pulse width has been created, which generates more intense X-rays than does the existing synchrotron radiation [21,22,23]. The serial femtosecond crystallography (SFX) or spectroscopic techniques using XFEL enables the determination of the molecular structures at room temperature without radiation damage [24,25,26]. Additionally, time-resolved SFX studies using an optical laser or mix-and-inject technique contribute to the making movies with high resolution molecular dynamics [15,27]. Moreover, due to advances in X-ray-focusing technology and detectors, time-resolved serial millisecond crystallography (SMX) studies as well as general serial synchrotron crystallography (SSX) are being performed at the existing synchrotron facilities [28,29,30]. Recently, the analysis of large complex structures using Cryo-EM technology has been in the spotlight [31], and the time-resolved EM technique is expected to be useful in the future for obtaining molecular dynamics information [32].

Additionally, computational molecular dynamics analysis by density-functional theory (DFT) or MD simulation provides an understanding of the functions of molecules and insights into their industrial or medical applications, such as in drug design [33,34,35,36,37].

Comprehensive molecular dynamics information for pinpointing the functions and properties of molecules can be inferred from information gleaned from multiple snapshots obtained using traditional techniques and the most advanced techniques. Examples of these include temporal characterization based on autocorrelation and pump probe technology combined with microscopy, spatial evolution analysis using X-ray and electron diffraction techniques, and monitoring of the temporal and spatial evolution of materials using nonlinear time-resolved spectroscopy [11].

In this special issue, we deal with the topic of molecular dynamics—those of small molecules to macromolecules. The issue includes a collection of comprehensive articles and reviews of research findings on a wide range of subjects related to molecular dynamics and the development of the technologies that makes its study possible. The results from this broad range of studies not only provide important insights about the technology but also facilitate a broader understanding of molecular dynamics studies across the various sciences.

## Figures and Tables

**Figure 1:**
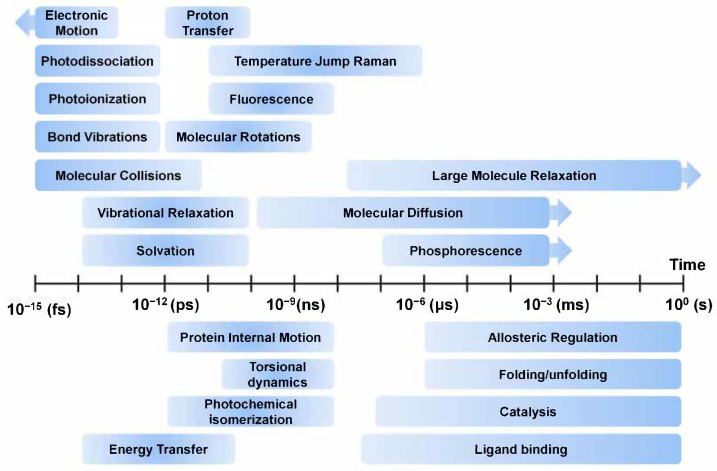
Overview of time scales of molecular motions. The contents of this figure were excerpted from the contents of the study mentioned in [8,9,10].

**Figure 2:**
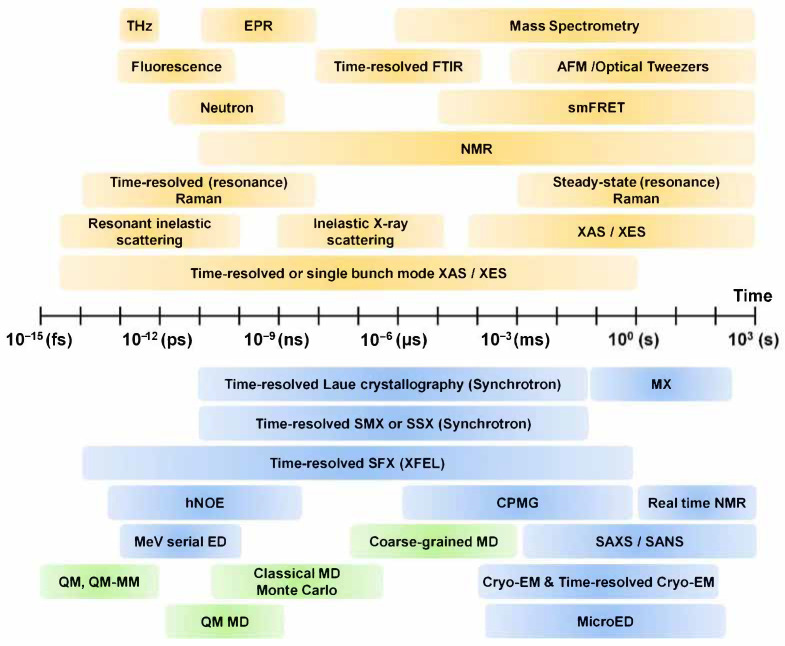
Techniques for structure and dynamics. THz: terahertz spectroscopy, EPR: electron paramagnetic resonance spectroscopy, FTIR: Fourier-transform infrared spectroscopy, smFRET: single molecule fluorescence resonance energy transfer, SMX: serial millisecond crystallography, SSX: serial synchrotron crystallography, SFX: serial femtosecond crystallography, hNOE: heteronuclear nuclear Overhauser effect, CPMG: Carr–Purcell–Meiboom–Gill, AFM: atomic force microscopy, ED: electron diffraction, SAXS: small angle X-ray scattering, SANS: small angle neutron scattering, MD: molecular dynamics, QM: quantum mechanics, MM: molecular mechanics. The contents of this figure were excerpted from the contents of the study mentioned in [15,16,17,18,19].
